# Prevalence of ESBL-Resistant Genes in Birds in Italy—A Comprehensive Review

**DOI:** 10.3390/ani15111598

**Published:** 2025-05-29

**Authors:** Muhammad Tahir Sarfraz Khan, Nicoletta Formenti, Giovanni Tosi, Flavia Guarneri, Federico Scali, Muhammad Kashif Saleemi, Eugenio Monti, Giovanni Loris Alborali

**Affiliations:** 1 Department of Translational and Molecular Medicine, Università degli Studi di Brescia, 25133 Brescia, Italy; eugenio.monti@unibs.it; 2 Istituto Zooprofilattico Sperimentale della Lombardia e dell’Emilia Romagna, 25124 Brescia, Italy; nicoletta.formenti@izsler.it (N.F.); giovanni.tosi@izsler.it (G.T.); flavia.guarneri@izsler.it (F.G.); federico.scali@izsler.it (F.S.); 3 Department of Pathology, University of Agriculture, Faisalabad 38040, Pakistan; drkashif313@uaf.edu.pk

**Keywords:** ESBL-producing bacteria, *Escherichia coli*, *Salmonella*, *Klebsiella*, ESBLs genes, *bla_CTX-M_*, avian species, phenotypic test

## Abstract

Antimicrobial resistance (AMR) is a major global concern in both human and veterinary medicine. Among antimicrobial resistance (AMR) bacteria, Extended-Spectrum Beta-Lactamases (ESBLs) pose a serious health risk because infections can be difficult to treat. These Gram-negative bacteria can be frequently found in poultry and in Italy, where such protein production is established. ESBL-producing *Escherichia coli*, *Salmonella* and *Klebsiella* in chicken and turkey may pose a significant public health risk due to potential transmission between poultry and humans. This review aims to assess the prevalence of ESBL-producing *E. coli*, *Salmonella* and *Klebsiella* phenotypically and genotypically in Italian poultry, identifying the most common genes, detection methods and potential information gaps. An initial pool of 1462 studies found in scientific databases (Web of Sciences, PubMed, etc.) was screened and 29 were identified as eligible for our review. Of these studies, 79.3% investigated both phenotypic and genotypic ESBL expression while ${bla}_{CTX-M}$, ${bla}_{TEM}$ and ${bla}_{SHV}$ were considered as targeted gene families. Large differences in prevalence were reported (0–100%). The ${bla}_{CTX-M-1}$ and ${bla}_{TEM-1}$ genes were the most prevalent in Italian territory. ESBL-producing *E. coli*, *Salmonella* and *Klebsiella* were frequently detected in farms and slaughterhouses, posing a potential threat to humans through contact (direct and indirect) with birds through handling, inhalation of infected dust, drinking contaminated water, ingestion of meat and meat products and the environment. Considering the frequent occurrence of ESBL-producing bacteria in Italian poultry, it is advisable to further improve biosecurity and to introduce more systematic surveillance. Additionally, the focus should be on the wild birds as they are ESBL carriers.

## Simple Summary

This review focuses on antimicrobial resistance (AMR), which is a great public health concern, and particularly on the Extended-Spectrum Beta-Lactamases (ESBL) in poultry in Italy. Three Gram-negative bacteria *E. coli*, *Salmonella* and *Klebsiella* can contribute to the spread of ESBL-resistance genes in poultry. These bacteria are involved in the transmission of resistance at farms and as well as slaughterhouses. The current study looks at the prevalence of ESBL-producing organisms in Italian poultry, the most frequent ESBL genes (such as blaCTX-M-1 and blaTEM-1), and the techniques used for identification. The number of isolates from *E. coli* and *Salmonella* are high, providing evidence of possible transmission between poultry, humans and the environment. The findings of previous studies underscore the importance of improved biosecurity, surveillance networks, and more studies on wild birds that may be carriers for such resistant pathogens.

## 1. Introduction

Antimicrobial resistance (AMR) poses a significant health risk worldwide for both human and veterinary medicine. Several studies have found that animal species can act as vectors or reservoirs for resistance genes [1]. Resistance in Gram-negative bacteria, particularly the *Enterobacteriaceae* family, can be acquired by plasmid-mediated horizontal transfer of antibiotic resistance genes [2,3]. Recent studies have shown that AMR in Gram-negative bacteria may be associated with the use of different antimicrobials even in a single treatment of certain classes (tetracyclines, potentiated sulphonamides, cephalosporins and macrolides) and the use of antimicrobials within the last 15–21 days of rearing [4,5,6].

In 2007, The World Health Organization (WHO) published the first report on critically important antimicrobials for human medicine [6] currently available is the sixth revision [7]. This report set priorities in the conservation of the efficacy of different classes of antimicrobials, in particular, the highest priority of critically important antimicrobials (HPCIAs) [8]. Among HPCIAs, some classes may be commonly used in animals, for example, third-generation cephalosporins. Extended-Spectrum β-Lactamase-producing (ESBL) bacteria can hydrolyse penicillin, cephalosporins (including the third generation) and aztreonam. They can also disassociate carbapenems and cephamycin [9]. Extended-Spectrum β-Lactamase-producing *Enterobacteriaceae*, such as *Escherichia coli*, *Salmonella* and *Klebsiella* spp. have been detected worldwide in humans, companion animals, livestock, food and the environment [9]. *Escherichia coli*’s potential of zoonotic relevance, whether from animals or food of animal origin, has been widely reported. In the last two decades, *E. coli*-producing ESBLs have been frequently documented in both animals and people, posing a severe risk to public health [10]. The WHO considers these bacteria as significant AMR pathogens and a public health issue [11]. In addition, the incidence of ESBL-producing *E. coli* in humans and animals, such as poultry, is growing worldwide [12].

There are nine different structural and evolutionary families of ESBLs, classified based on amino acid sequence including

${bla}_{TEM}$ (Temoniera Extended-Spectrum Beta-Lactamase)

${bla}_{GES}$ (Guiana Extended-Spectrum Beta-Lactamase)

${bla}_{SHV}$ (Sulfhydryl Variable Extended Beta-Lactamase)

${bla}_{CTX-M}$ (Cefotaxime-Mediated Extended Beta-Lactamase)

${bla}_{OXA}$ (Oxacillinase Extended Beta-Lactamase)

${bla}_{VEB}$ (Vietnamese Extended-Spectrum Beta-Lactamase)

${bla}_{TLA}$ (Tlahuizcalpan Beta-Lactamase)

${bla}_{BES}$ (Brazilian Extended-Spectrum Beta-Lactamase)

as reported by this study [13]. The *bla_TEM_*, *bla_CTX-M_*, *bla_SHV_* and *bla_OXA_* are the major groups that have been identified as ESBL resistance genes [14]. The *bla_TEM_*, the first β-lactamase found in ESBL bacteria, is thought to have originated in *E. coli* [15]. In poultry and humans, the most common ESBL gene is *bla_CTX-M_* and the variants of this ESBL gene vary in both species [15,16]. The transmission of an antibiotic-resistant plasmid, pSL222-6, in *E. coli* from hens to human handlers was described as far back as the early 1970s [17]. Furthermore, it has been reported that *bla_CTX-M_*, *bla_CTX-TEM_* and *bla_CTX-SHV_* families, which are poultry-associated genes mostly found on plasmids, are also present in humans [18,19]. The spread of pathogenic bacteria between poultry and humans was also suggested when the beta-lactamase *bla_CTXM-15_* and its closely related ESBL genes were identified in *E. coli* isolates from poultry and humans [20,21]. In addition, the plasmids involved in ESBL production have the potential to cause resistance to other antimicrobials, including fluoroquinolones, trimethoprim and aminoglycosides, because they can carry their respective resistance genes. As a result, it would be difficult to treat diseases caused by ESBL-producing bacteria such as *E. coli*, *Salmonella* and *Klebsiella*. With one health prospective, the spread of ESBL plasmids in the bacterial population could exacerbate this problem in humans, animals and the environment [22,23].

Excessive antimicrobial use (AMU), which had been verified through antimicrobial susceptibility testing of various *Salmonella* samples from the food chain (2016–2019) in Italy, has the potential to enhance the spread and selection of AMR bacteria [24,25] and thus reducing the effectiveness of antimicrobials for human and veterinary medicine as a result posing a great challenge for one health. Italy is one of the highest consumers of antimicrobials in livestock within the European Economic Area (EEA), but there has been a steady decline in recent years [26]. This reduction seems to be particularly evident in the poultry sector, where a study on a large sample of farms, representing approximately 30% of Italian production, showed a decrease of 71% in AMU in broilers and 56% in turkeys over three years (2015–2017) [27]. Furthermore, the largest association of the poultry sector (UNAITALIA), which implements the plan for almost the entire national production, approved a plan where it was made an obligation to adopt electronic prescriptions and the collection of data in the classy farm system, and reported a 94% decrease of usage use in chickens and 92% decline in the antibiotic use in 2022 as compared to 2015 [28]. The prevalence of ESBL-producing *E. coli* seems to be decreasing between 2016 and 2022, it fell from 94 to 47% in broilers, from 78 to 47% in turkeys and from 68 to 40% in broiler meat [29]. Despite all these efforts and the withdrawal of third- and fourth-generation cephalosporins in the Italian poultry sector for more than a decade, those prevalences are still very high, and there is a need to continuously improve the surveillance of these bacteria, especially considering the complexity of ESBL epidemiology [30]. Therefore, it is essential to study the prevalence of AMR in poultry and other animals by characterising resistance genes and also considering plasmids [31,32].

### Impact of Antibiotics Usage in Poultry Industry and One Health Approach

Antibiotic use and abuse has been identified and enriches antibiotic-resistant microorganisms in the gastrointestinal microbiome of feeding animals, especially broilers [33]. Thus, AMR bacteria may be present in the intestine of adult breeder hens and shed the bacteria in their faecal contents resulting in the contamination of the eggs’ outer surface and shell, leading towards the transfer of resistance genes in the environment [34]. The hatching of infected eggs (egg shells) by pathogenic bacteria results in a poor and adversely affected health status of birds and their meat products and byproducts [35]. The antimicrobial resistance (AMR) bacteria which are residing and emerging in the farming system can transfer from humans through animal–human interaction or the consumption of animal products or contact with meat products. Although heat treatment can remove viable AMR bacteria, it may not completely degrade bacterial DNA; consequently, antimicrobial resistance genes can still be detected using molecular approaches like PCR [36,37,38]. Additionally, there is chance of cross contamination when an uncleaned instrument is applied, or the rupturing of the gastro-intestinal tract (GIT) occurs during animal slaughtering or food processing time, resulting in the transmission of AMR bacteria in the environment [39]. An examination of 638 retail meat samples, including beef, pig, and poultry, indicated that 109 (17.1%) contained ESBL-producing *E. coli.* Notably, poultry meat had the highest contamination rate, with 60 of 198 samples (30.3%) testing positive in Portugal [40]. The use of antibiotics is the most vital factor involved in the selection pressure which enables the spread of AMR genes, and unluckily, it is not only antibiotic consumption that causes antibiotic exposure [41,42]. There are several other factors which contribute in the spread of resistance genes and bacteria. It is estimated that 90% of the antibiotic’s doses excreted partially metabolised or unchanged through faeces and urine after administration [43]. However, animal waste utilisation as fertiliser in crops makes the environment susceptible to antibiotic residues [44]. The bacteria resistance could be shifted to the wild once it is present in the environment, particularly in migratory raptors and wild birds, those that have the potential of traveling a long distance through various niches of ecological nature and pray on small birds and are synanthropic in rural and urban areas [35,45].

This review aims to evaluate the current literature on ESBL-producing *E. coli*, *Salmonella* and *Klebsiella* in the Italian poultry production chain, to assess the phenotypic and genotypic prevalence, the most common ESBL genes and commonly used detection methods. It will also be identifying potential gaps of knowledge regarding ESBL in the Italian poultry industry.

## 2. Materials and Methods

Four databases were considered for the literature research including Google Scholar, PubMed^®^ and Science Direct, Scopus and Research Gate from (1 January 2001 to 31 December 2024), applying different combinations of words such as “ESBL-Producing bacteria” in “Poultry” in “Italy”, “ESBL-encoding bacteria” and “Poultry” in “Italy”, “*Enterobactiaceae*-encoding ESBL” in Italian territory in “Poultry birds”, ‘‘ESBL-*Klebsiella*-poultry’’, “Extented-Spectrum-Beta-Lactamase-producing *Salmonella*” in “Italy” in “Avian species” and “birds” “ESBL resistance in birds in Italy” (Supplementary Materials Table S1).

Various websites of different organisations were also searched to identify the relevant literature required, including the World Organisation for Animal Health (WOAH, founded as OIE), the International Livestock Research Institute (ILRI), the Food and Agriculture Organization of the United Nations (FAO), the World Health Organization (WHO), the World Food Bank, the World Bank, the European Union, the European Nation Antibiotic Control Programme, National Plan to Contrast Antibiotic Resistance (Piano Nazionale di Contrasto all’Antibiotico-Resistenza—PNCAR and the National Plan to Contrast Antibiotic Resistance (Piano Nazionale di Contrasto all’Antibiotico-Resistenza—PNCAR) [46] and Reference Centres, details presented in Table S1.

### 2.1. Data Inclusion Protocol

This review included the following search data:

1. Observational studies in which *E. coli*, *Salmonella* and *Klebsiella* spp. producing ESBL phenotypically and genotypically associated with Extended-Spectrum Beta-Lactamases were detected in cloacal swabs, caecal contents, faecal samples and some chicken and turkey meat and food samples, including samples from abattoirs, farms and food markets.
2. All studies were carried out in Italian public institutes, universities and national reference centres for antimicrobial resistance. The data published in English between 2001 and 2024 were included, and data related to ESBL, where different gene resistance patterns, transfer patterns and plasmid and transfer patterns were discussed, were extracted.
3. The review included only data from studies on domestic and commercial poultry and their meat.
4. Studies related to broiler, breeder, layers and turkey were our focus and included in our review

### 2.2. Data Exclusion Protocol

This review set the following criteria for exclusion:

1. Studies involving bacterial species other than *Salmonella, E. coli* and *Klebsiella* spp. or specific strains of *Salmonella* and *E. coli* that are unable to produce or carry genes for ESBL.
2. Studies that focused on the environment and animal species except poultry.
3. Studies that investigated only geese, ducks, parrots, pigeons and wild bird species.
4. Studies which included samples from water and dust samples within poultry areas, litter material, samples from eggs in hatchers and setters and sampling from egg storage rooms because these samples mainly associated with the environment.
5. Abstracts of conferences, chapters from textbooks and books and case studies where full text was not available. In Supplementary Materials, Table S3: list of the excluded studies and the reason for their exclusion is provided.
6. This lack of early data before 2000 is in line with findings reported in the literature, which show that systematic monitoring of ESBLs in food animals in Europe began predominantly in the early 2000s [47].

### 2.3. Screening of Studies

**Figure 1:**
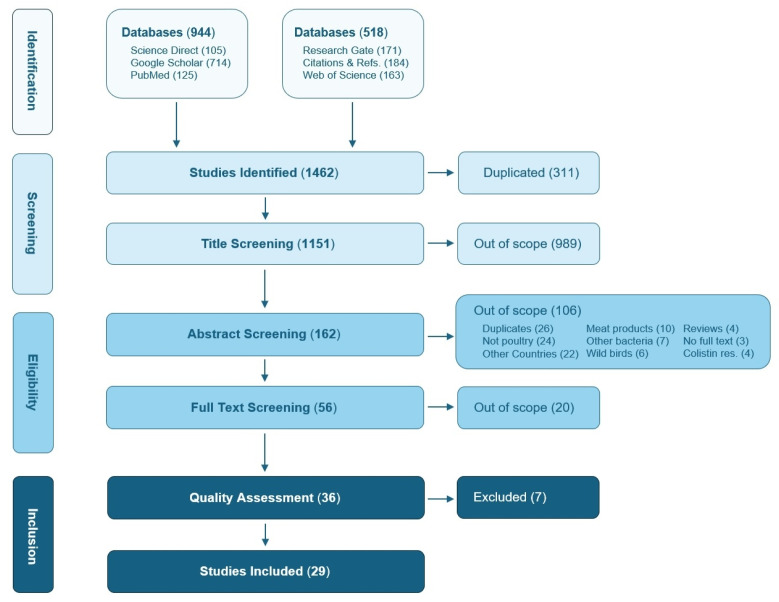
Prisma flow chart used for the screening process [48].

All the studies identified applying the search protocol, according to the PRISMA standards, were sent to the reference management software Zotero 6.0.26 (Corporation for Digital Scholarship, Vienna, VA, USA) and all duplicated studies were excluded. By following the eligibility criteria, screening of specified studies was carried out in three steps including, abstract, title and full-text levels as shown in Figure 1.

Tracking of references and citations of eligible published studies was performed to identify past unrecognised research studies related to our systemic review. This protocol was adopted together with the data extraction process.

### 2.4. Extraction of Data

A data extraction template was created using Microsoft Excel 365 (Microsoft Corporation, Redmond, WA, USA). Column headings were defined based on the eligibility criteria and research questions. The data we examined included general publication details (publication date, country, authors and study period), study specifications (sampling plan, sample size, study design), research questions and objectives, species, age, production system), study outcome (techniques used for bacterial identification, phenotypic diagnosis of ESBL-encoding bacteria, identification of ESBL genes, percentage and frequency of ESBL) and ESBL-producing bacterial species in the study. Percentage (%) of ESBL refers to the number of *E. coli*, *Salmonella* and *Klebsiella* spp. producing ESBL out of the total number of samples of all three organisms tested. The production systems were also reported according to the studies of different authors. The systems include small-scale, backyard, extensive, semi-intensive and intensive systems.

### 2.5. Data Analysis

In this review, we were not able to perform a meta-analysis because of the heterogeneity of the data. Instead, we used figures and tables to provide a narrative analysis of the available study specifications. We grouped the studies into three geographical areas: Southern, Central and Northern Italy, according to the national geographic scheme.

## 3. Results

Overall, 1462 studies were identified through the scientific databases from January 2001 to December 2024. The number of studies found for each database is reported in Figure 1. Through full text, abstracts and titles, and a two-tier screening process, 29 research studies were considered eligible for inclusion in our review.

A Prisma flowchart summarising this screening is shown in Figure 1.

### 3.1. Study Quality

Most of the studies (22 out of 29, 75.86%) included in this review did not perform probabilistic sampling, thus they failed to meet the criteria for bias, such as proper sample size and sample type, and were rated as of moderate quality, corresponding to a moderate risk of bias. Only a few studies met the criteria for high quality as they provided adequate information on sample size, species identification, phenotypic and genotypic identification methods used in the research, used appropriate statistical analysis and the conclusions were consistent, relevant and logical with the results of the studies.

**Figure 2:**
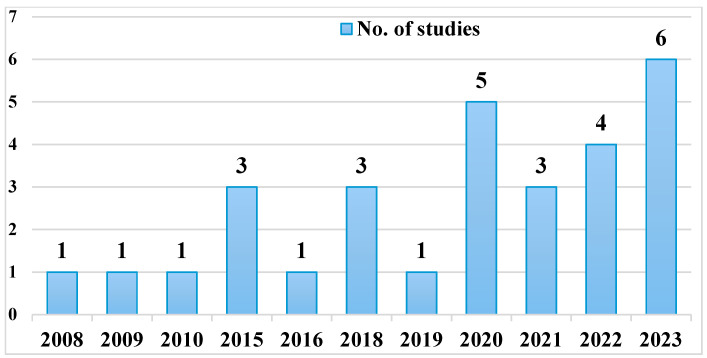
Three out of 29 studies (10.3%) collected samples in year 2015, 2018 and 2021 from different areas of the country. In terms of temporal distribution, years 2020, 2022 and 2023 were the years in which the most studies were conducted: 5 (17%), 4 (13.7%) and 6 (20.6%), respectively. Y axis shows the distribution of studies between 2000 and 2024.

**Figure 3:**
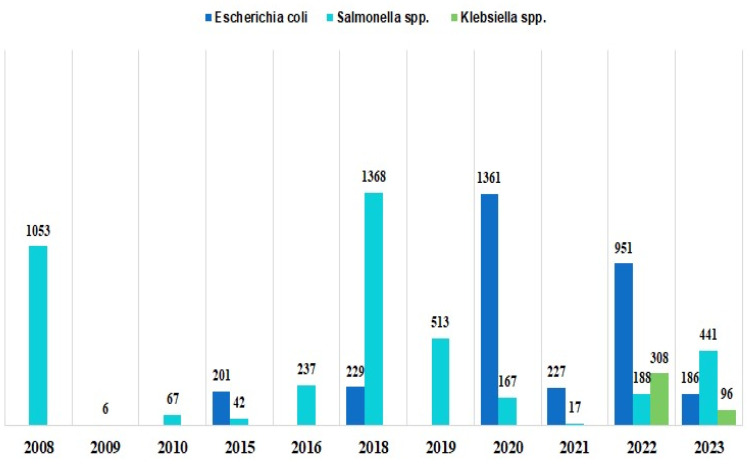
A total of 3155 *Eschresia coli* (*E. coli*), 4099 *Salmonella* spp. and 404 *Klebsiella* spp. samples were collected in the 29 studies from all of Italy. The sample size varied considerably among studies for both *E. coli* (median 186; range 33–855) and *Salmonella* (median 612; range 6–1053) and *Klebsiella*, with only 2 samples (96 and 308).

Most studies (26 out of 29, 89.6%) were carried out in only one of the three main geographical areas of Italy: 9 (31%) in Northern Italy, 11 (37.9%) in Central Italy and 6 (20.1%) in Southern Italy as indicated in Figure 2. It is reported that the from last ten years major studies were conducted, and most samples were taken, most samples (1597) were collected in year 2018 followed by (1528) in year 2020. While least samples (6) were collected in year 2009 as shown in Figure 3.

### 3.2. Sampling Strategy and Study Design

Almost all the studies were cross-sectional investigations. There was no comparative study conducted in these available articles as shown in Table 1.

**Figure 4:**
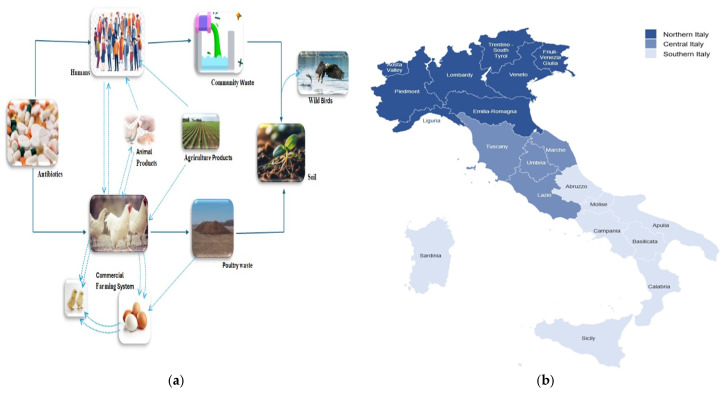
The figures indicate (**a**) a flow diagram showing the cycle of antimicrobial resistance (AMR) transmission within poultry, human, agricultural settings and wild birds; (**b**) the map of Italy indicating the regions e.g., Northern, Central and Southern.

This diagram depicts the interrelated pathways for antibiotic-resistant bacteria (AMR) transmission in a commercial chicken farming system. Antibiotics used in agriculture trigger the development of resistance, which is distributed through animal products, waste from poultry, agricultural procedures, and community waste, eventually affecting humans and wildlife. The figure emphasises the relationship between humans, animals, and the surrounding environment in the AMR cycle as shown in Figure 4a.

### 3.3. Nationwide Studies

Three out of 29 (10.3%) studies were carried out at the national level [49,50,51] in Italy. ESBL resistance was detected in two bacteria, *Salmonella* [49,50] and *E. coli* [51]. The samples were collected from slaughterhouses and two studies were carried out at farms. The Kaufman serotyping method was applied to identify *Salmonella* spp. A matrix-assisted laser desorption ionisation (MALDI) time of flight (TOF) mass spectrometry (MS) analysis was carried out to identify *E. coli*. Phenotypic and genotypic tests, along with WGS [51], were performed in all studies conducted nationwide. In the national surveillance studies, microdilution and disk diffusion methods for antibiotic susceptibility testing along with phenotypic identification of ESBL were applied to investigate AMR [49,50]. All these studies depicted multi-drug resistance (MDR) bacteria prevalence in Italy. The guidelines of the European Committee for Antimicrobial susceptibility testing (EUCAST) were followed by [50,51] and both the Clinical and Laboratory Standards Institute (CLSI) and EUCAST in one study [50]. Only two (6.89%) ESBL-resistant genes, *bla_CTX-M-1_* and *bla_CTX-M-15_*, were detected in national studies. One study [50] has described plasmid types IncX4 and IncF11 from *Salmonella* species.

### 3.4. Nothern Italy

Nine out of twenty-nine (31%) studies were conducted in Northern Italy [52,53,54,55,56,57,58,59,60]. The PCR [52,53,54,55,56,57,58] and Indol test [58], β-Glucuronidase test [53] and serotyping [56] were used in five (55%) studies while WGS was performed in one study for identification of species. Three out of nine studies (33.3%) investigated antimicrobial susceptibility using the disk diffusion method [52,55,56], only one study used the broth microdilution test [53]. Four (44%) studies performed the double disk synergy method [54,55,58,60] and two studies out of nine (22%) applied the microdilution method for phenotypic identification of ESBL-resistance [56,57].

Six studies (66%) detected ESBL genes from *E. coli*, and one study (11.11%) identified ESBL from *Klebsiella* spp. [59] and two studies (22.22%) found ESBL-resistant genes from *Salmonella* species [53,56].

In our selected studies, isolates obtained from most of the studies showed multi-drug-resistance (MDR), but two studies did not investigate the MDR [56,60]. The most common ESBL gene was the CTX family (*bla_CTX-M-1_*, *bla_CTX-M-15_*, *bla_CTX-M-55_*) which was detected in five out of nine (55.55%) [52,53,54,59,60] studies while three (33.5%) studies described *bla_TEM_* [55,57,58], while four studies (44.4%) detected *bla_SHV_* [54,55,56,59,60]. The plasmids identified are IncK2, IncI1, IncX3, IncF1B and IncF11 [59], IncI1, IncN and IncF1B [55] Inc1_1γ_, IncI-1, IncK, IncF1B and IncN [52]. Plasmid types were identified in the northern Italian region. Another resistant gene *bla_CMY-2_* was also detected in one study only [60].

### 3.5. Central Italy

Eleven out of twenty-nine (37.9%) studies in Central Italy were conducted in the region of Tuscany [61,62,63,64,65,66,67,68,69,70,71]. Four out of eleven (36.4%) studies investigated ESBLs in *E. coli* [62,68,70,71], only one study (9.1%) [69] focused on *Klebsiella* spp., while seven studies (63.6%) focused on *Salmonella* [61,63,64,65,66,67]. Note: One study [67] investigated the isolates from both *E. coli* and *Salmonella*. A selective media, MacConkey agar was utilised to grow ESBL bacteria in four studies (34.3%) [61,63,68,70]. The confirmation tests for the bacterial species were performed using PCR [61,68,70], biochemical [63,71] and serotyping analyses [68]. One study did not perform bacterial confirmation tests, phenotypic tests and antibiotic susceptibility tests as they directly performed whole genome sequencing (WGS) and identified the ESBL genes and plasmids responsible for the resistance and transfer of genes [67].

One study did not proceed with the isolates of *E. coli* for the genotypic analysis of ESBL resistance genes; they performed only phenotypic analysis as indicated in Table 1 [62]. The most common ESBL gene was *bla_CTX-M-1_*, detected in six studies (54%), after this *bla_TEM_* was identified in four studies (34.3%) [65,66,68,71]. Other ESBL genes were *bla_CTX-M-15_* [67,69,71] *bla_SHV_* [71] as shown in Table 1. The disc diffusion method was the most commonly used (seven out of nine studies) to determine susceptibility [61,62,65,66,69,70,71]. However, three studies [63,64,68] applied the microdilution test as indicated in Table 2. Only one study reported data on antimicrobial usage (AMU), which is regarding amoxicillin and doxycycline [71].

Six studies (54%) reported the presence of multidrug-resistant (MDR) strains among identified bacterial species through phenotypic testing [61,62,63,68,70,71]. Several plasmids were identified in different studies in Central Italy, as complete details are available in Supplementary Table S2: IncX [65,67] IncX1, IncX4 [63,65] IncF1, IncF11, IncH12, IncI and IncN [68,69], which have been identified in the included studies while the most common plasmids were IncX, IncI, IcF and IncN families.

### 3.6. Southern Italy

Six studies out of twenty-nine (20.7%) were conducted in Southern Italy [72,73,74,75,76,77]. Three studies (50%) investigated the Extended-Spectrum Beta-Lactamases resistance in *Salmonella* isolates [73,76,77]. Another three studies (50%) identified ESBL genes from *E. coli* isolates in Southern Italy, as shown in Table 1 [72,74,75]. In two studies (33.3%), only a phenotypic investigation of ESBL resistance was performed, and authors did not perform genotyping analysis [72,76]. Samples were collected: one from the market [72], three from the farms [73,74,77] and two studies did not report the sampling background, as shown in Table 1 [75,76]. The double disc synergy test for phenotypic identification was used in two studies [74,75]. Other studies did not report any phenotypic confirmation tests. The Kirby–Bauer method was used for antimicrobial susceptibility testing in one study [76], the broth microdilution method [73] and the disk diffusion method were applied in two studies [72,75], as presented in Table 2. The most common gene was *bla_CTX-M-1_*, which was detected in three studies [74,75,77], while the *bla_SHV_* gene was detected in the Southern Italian poultry industry in only one study [73]. Other ESBL-related genes were *bla_CTX-M-15_* and *bla_TEM_* families, while two studies [72,76] did not investigate the ESBL-resistant genes as indicated in the Table 1. Four out of six (67%) studies used EUCAST and two (33%) used CLSI as cutoff values and phenotypic identification guidelines, as shown in Table 2. None of the studies reported data on AMU. Two studies (33.3%) applied whole genome sequencing for plasmids and resistant genes investigation and identified IncX1-4, IncF1B and pESL-like plasmids in *Salmonella* isolates as shown in Table S2.

### 3.7. Extended-Spectrum Beta Lactamase Identification Methods

Three studies were carried out on slaughterhouse isolates [48,54,74]. All other studies were performed on samples collected from poultry farms and markets, according to the map, provided in the Figure 4b. Sampling was carried out using meat, faecal, cloacal, skin and chicken meat products, while broiler chicken, layer and turkey were under consideration only as indicated in Table 1.

Twenty-three out of twenty-nine (79.3%) studies reported both phenotypic and genotypic characterisation of ESBL, three studies (10.3%) reported phenotypic characterisation only [62,72,76] and three studies (10.3%) reported genotypic characterisation only, as shown in the Table 1 [60,67,74]. Thirteen studies (44.82%) identified ESBL-resistant genes from *E. coli* and the other 13 (44.82%) from *Salmonella*, one study used *E. coli* and *Salmonella* isolates [68] while two studies (6.9%) identified resistance from *Klebsiella* spp. [59,69]. There were 12 studies (41.3%) which employed WGS, while 5 studies (17.2%) performed restriction fragment length polymorphism (RFLP) [50,52,56,57,78] for the identification of resistant genes. The PCR was performed in 22 (75.86) studies conducted in Italy to investigate the ESBL-resistant genes in Gram-negative bacteria, as shown in Table 1.

Twelve studies (41.2%) reported the application of the disk diffusion test for antibiotic susceptibility testing, nine studies (31%) employed the broth microdilution method and only one study [62] used both methods. It has been evidenced that only one study [69] applied the Kirby–Bauer method, and one study [68] worked with the VITEK^®^ 2 system for the phenotypic testing of isolates collected from Italy, as indicated in Table 2. Six studies (23%) did not consider antibiotic susceptibility testing and employed only genotypic identification methods.

It was reported that 12 (41.3%) selected studies applied CLSI, and 12 (41.3%) studies used EUCAST, and two (6.89%) studies [49,57] used both CLSI and EUCAST guidelines to perform the phenotypic tests in Italy. However, three studies did not adopt the phenotypic identification method [60,67,74].

The commonly used methods for phenotypic confirmation of ESBL strains were the double disc synergy method in nine studies (31%) and the broth microdilution method in seven studies (24%), while only one study (3.44%) applied the VITEK^®^ 2 system [68]. On the other hand, 12 studies (41.37%) did not perform phenotypic confirmation tests at all. Our findings identified only three studies (10.34%) that only considered genotypic methods.

ESBL genes such as (*bla_SHV_*, *bla_TEM_* and *bla_CTX-M_*) were identified through PCR and WGS analysis in Italian studies. However, the RFLP was also employed to detect the genotype of ESBL-resistant bacteria. Most of the studies confirmed the gene-specific ESBL families while few studies only specified the CTX-M-1, TEM gene family.

Details of all phenotypic and genotypic identification methods used by the included studies are reported in Tables S1–S3, while the excluded studies are reported in Table S3 in the Supplementary Materials.

## 4. Discussion

The 29 studies eligible for this review were too heterogeneous, using different methods for both phenotypic and genotypic analyses of ESBL resistance. The prevalences varied widely, in the range of 0–100%, with differences also among areas. Twenty-three out of the twenty-nine studies (79.3%) confirmed ESBL production by both phenotypic and genotypic methods, while the other twelve (20.7%) used either phenotypic or genotypic methods only. The *bla_CTX-M_*, *bla_SHV_* and *bla_TEM_* were the most studied genes, with *bla_CTX-M_* being the most frequently found. However, the frequency of identification varied between Italian regions. This could be due to differences in sample size, sample origin (farm, slaughterhouse, meat, etc.), detection methods, production system and biosecurity level. In addition, most studies used convenience samples (non-random, non-probability sampling), which makes it even more difficult to assess the prevalence of these genes.

Regarding the bacterial species, there seems to be no major differences in the distribution of ESBL genes between *Salmonella* and *E. coli* and *Klebsiella* spp. Many ESBL encoding genes including *bla_CTX-M_*, *bla_SHV_*, *bla_TEM_*, *bla_GES_*, *bla_IRT_*, *bla_BEL_*, *bla_PER_*, *bla_VEB_*, *bla_TLA_* and *bla_CMT_* [79,80] are reported in several studies throughout Italian territories and several other genes were not reported. However, most studies have reported that these bacteria carry only three main ESBL genes: *bla_TEM_*, *bla_CTX_* and *bla_SHV_*. The other families of genes encoding beta-lactamases, such as *bla_GES_*, *bla_BEL_*, *bla_IRT_*, *bla_PER_*, *bla_TLA_*, *bla_VEB_*, *bla_CMT_* and *bla_BES_* were rarely or never detected in Italy. This may be due to the chromosomal rather than plasmidic location of these genes [9,81]. Among these families, *bla_GES_* seems to be the most common of the minor ESBL [82]; it was first reported in *Enterobacteriacae* [83], but it seems to be most common in *Pseudomonas aeruginosa* and *Acinetobacter baumannii* [82] not in the Gram-negative bacteria. The ESBL gene *bla_VEB_* was initially identified in *E. coli* [84], and later reported in several Gram-negative bacteria [85]. The *bla_PER_* and *bla_BEL_* were firstly described in *P. aeruginosa* [86], and later found in other bacterial species, including *Enterobacteriacae* [81,87,88]. Although *bla_GES_* and *bla_VEB_* have been reported in Europe, only *bla_PER_* and *bla_BEL_* have been found in Italy [82,89]. The *bla_TLA_* and *bla_BES_* seem to be restricted mainly to Mexico and South America regions and less common in Italy.

The frequent detection of *bla_CTX-M_*, particularly the *bla_CTX-M-1_* and *bla_CTX-M-15_* variants, is consistent with other studies conducted in Africa, Asia, Europe and the Americas, where ESBL-resistant genes were identified in the poultry [90,91,92,93]. The most common ESBL gene is *bla_CTX-M_* and the variants of this ESBL gene vary in different settings [25,94]. Indeed, recent research has reported that *bla_CTX-M_*-type enzymes appear to be the current dominant ESBL-type present in several regions worldwide [9,95]. The *bla_SHV_*, *bla_TEM_* and *bla_CTX-M_* are the most common ESBL resistance genes in several countries globally including Europe, Asia, South and North America and Africa continents as reported in a study by Tseng et al., 2023 [25,96]. The dominant ESBL gene in Asia is *bla_CTX-M-__15_*, while Europe is also facing the problem of *bla_CTX-M-1_* resistance prevalence in poultry [91]. The dominant ESBL gene in the European poultry industry seems to be *bla_CTX-M-_*_1_ which are similar to our findings in this in Italy.

In our review, the presence of ESBL genes such as *bla_SHV_* and *bla_TEM_* families were accordance with what has been reported in a previous Asian study [97]. No studies detected *bla_OXA_* genes from *E. coli* in poultry in Italy and this may be due to a species-specific character, that the *bla_OXA_* gene is identified in *Pseudomonas autogiros* and other species [98,99], not in *E. coli*, *Salmonella* and *Klebsiella* spp.

In recent times, plasmids carrying ESBL genes of *E. coli* have emerged as a major concern globally; horizontal transmission potential of these plasmids among different host species, including humans and animals, and within different bacterial species, poses a great risk for humans by means of direct contact or horizontal transmission [100,101]. In fact, plasmids play a vital role in the spread of ESBL-encoding genes between humans and other animals. Several Italian studies on *E. coli* reported the presence of plasmids carrying beta-lactamase genes (IncX, IncI, IncF, IncK, IncN, etc.) in isolates from poultry, cattle, camels and goats [102,103,104,105] which also justify our findings. In addition to plasmids, the spread of beta-lactamase genes is associated with the exchange of genome particles and the spread of bacterial clones encoding ESBL genes. All these phenomena are highly variable, making it difficult to identify the sources and routes of transmission of ESBL genes in bacteria as our review highlights the same results [106].

Our study identified that the PCR with specific primers 23 (79.31%) for the beta-lactamase genes was the simplest and most widely used molecular method to identify the genes encoding these enzymes and their specific families. The primers were selected by identifying the specific region to be annealed without evidence of mutation [14,83]. Nevertheless, evidence suggests that the use of different primers for gene sequencing may increase the variability of results, making comparisons even more limited [107]. Similarly, in our review we identified that there were limited data available on the use of antibiotics at poultry farms; this may be due to the ban on the use of antibiotics in poultry from the last decade. Although data on certain ESBL genes were available for several Italian administrative regions, others were still not covered, such as Piedmont, South Tyrol, Sardinia, and Liguria. Data on backyard poultry production were also not available. Further investigations are needed in different regions and for layer and backyard poultry to obtain more data on the distribution of ESBL-encoding bacteria in poultry. This information should help to verify the effectiveness of current strategies. These include the drastic reduction of AMU in the Italian poultry sector, the banning of certain cephalosporins, the use of alternatives to antimicrobial therapy, increased awareness among stakeholders and the promotion of hygiene in husbandry practices.

In our study, we detected ESBL genes from Gram-negative bacteria (*E. coli*, *Klebsiella* spp., *Salmonella*) in poultry only. There are several studies where Extended-Spectrum Beta-Lactamases genes were identified from the wild and migratory birds, especially in the southern part of Italy [108,109] from *Klebsiella* spp., which poses a great risk for poultry as well as humans, as the transmission from the wild is very difficult to control [110,111]. Only strict biosecurity measures could be effective in reducing the transmission between poultry, wild birds and humans. Further studies are required to investigate the prevalence as well as the transmission of the ESBL genes from wild birds to poultry, animals and humans.

There are some important limitations to this review. Firstly, only a limited number of bird species were considered, namely broilers, layers and turkeys. Secondly, the review used a narrow set of keywords and excluded the literature written in Italian, German and French. Few samples from the environment which were directly or indirectly associated with poultry settings were excluded because of uncertainty about available data. Additionally, we only focused on three Gram-negative bacterial species of *Enterobacteriaceae*. At the end, data from certain Italian regions were missing which may be due to a lack of funds or facilities but we could not find the exact reason. Unfortunately, these limitations may have led to the overlooking of useful data published in articles that did not meet the strict inclusion criteria.

## 5. Conclusions

The prevalence of ESBL-producing *Salmonella*, *E. coli* and *Klebsiella* spp. in the poultry population on the Italian territory poses a potential threat to both humans and livestock in terms of horizontal transmission or direct or indirect contact with the birds or their products. These bacteria may pose a zoonotic risk to the human population, through the consumption of poultry meat and products, but also through direct physical contact with birds. The synthesis of the available data showed a wide distribution of ESBLs producing *Salmonella*, *Klebsiella* and *E. coli* at various levels in Italy and the most frequently detected genes were *bla_CTX-M_*, *bla_TEM_* and *bla_SHV_*. However, the data available from the current studies did not allow their prevalence to be estimated with sufficient precision, highlighting the need for comprehensive and standardised surveillance in the country, also considering the major efforts to reduce and rationalise AMU in the Italian poultry sector. To better understand the distribution and diversity of ESBLs in bacterial populations and their impact on public health, standardised surveillance protocols in poultry, wild birds and other farm animals should be adopted.

## Figures and Tables

**Table 1:** Summary of the characteristics of the 29 studies included in the review on Extended-Spectrum Beta-Lactamases (ESBLs) in *Escherichia coli*, *Salmonella* and *Klebsiella* isolated from poultry from different areas of Italy.

Sampling Area	Sampling Site	Sampled Species	Sample Source	Sample Size	Health Status	Bacterial Species	Detection Method	Genes	Reference
Nationwide	Slaughterhouses	Chicken	Carcass, Meat Products	85	Diseased	*Salmonella* spp.	Phenotypic, PCR ***, WGS *	*bla_CTX-M-__1_*	[49]
	Farms	Chicken	Faecal, skin, liver, meat products	87	Diseased	*Salmonella* spp.	Phenotypic, PCR ***, RFLP **	*bla_CTX-M-1_*	[50]
	Farms	Chicken, pigs, cattle	Faecal, caecal, intestinal contents	194	Diseased	*Escherichia coli*	Phenotypic, PCR ***, WGS *	*bla_CTX-M-15_*	[51]
Northern Italy	Farms	Chicken	Cloacal swab	229	Diseased	*Escherichia coli*	Phenotypic, PCR ***, RFLP *	*bla_CTX-M-1_*	[52]
	Farms	Chicken	Faecal	6	Healthy	*Salmonella* spp.	Phenotypic, PCR ***	*bla_CTX-M-1_*	[53]
	Slaughterhouses	Chicken	Carcass, faecal, cloacal swabs	513	Diseased	*Escherichia coli*	Phenotypic, PCR ***	*bla_CTX-M-1_*, *bla_SHV_*	[54]
	Farms	Chicken	Faecal	67	Healthy	*Escherichia coli*	Phenotypic, PCR ***, RFLP **	*bla_TEM-1_*, *bla_SHV_*	[55]
	Farms	Chicken	Faecal, meat products	1053	Diseased	*Salmonella* spp.	Phenotypic, PCR, RFLP **	*bla_SHV-12_*	[56]
	Mixed	Chicken	Carcass, meat	33	Diseased	*Escherichia coli*	Phenotypic, PCR ***, WGS *, RFLP **	*bla_TEM_*	[57]
	Farms	Chicken	Carcass, meat	142	Diseased	*Escherichia coli*	Phenotypic, PCR ***	*bla_TEM_*	[58]
	Farms	Chicken	Rectal swabs	308	Healthy	*Klebsiella pneumoniae*	Phenotypic, WGS *	*bla_CMX-__15_*, *bla_SHV-__27_*	[59]
	Farms	Chicken	Cloacal swabs	100	Diseased	*Escherichia coli*	WGS *	*bla_CMY-2_*, *bla_CTX-M-65,55_*	[60]
Central Italy	Mixed	Chicken	Faecal	42	Healthy and Diseased	*Salmonella* spp.	Phenotypic, PCR ***, WGS *	*bla_CTX-M-1_*	[61]
	Farms	Chicken	Skin, cloacal swabs	406	Healthy	*Escherichia coli*	Phenotypic	ND	[62]
	Farms	Chicken, turkey	Faecal, caecal	1044	Diseased	*Salmonella* spp.	Phenotypic, PCR ***	*bla_CTX-M-1_*	[63]
	Farms	Chicken	Carcass, meat	324	Diseased	*Salmonella* spp.	Phenotypic, PCR ***, WGS *	*bla_CTX-M_*	[64]
	Mixed	Chicken	Meat products	80	Diseased	*Salmonella* spp.	Phenotypic, PCR ***, WGS *	*bla_CTX-M-1_*, *bla_TEM_*	[65]
	Farms	Chicken, pigs	Insect contaminated Meat	105	Diseased	*Salmonella* spp.	Phenotypic, PCR ***	*bla_TEM_*	[66]
	ND	Chicken	Meat	5	Diseased	*Salmonella* spp.	WGS *	*bla_CTX-M-1_*, *bla_CTX-M-15_*	[67]
	Farms	Chicken	Carcasses, Litter	180 + 6 *	Healthy	*E. coli*, *Salmonella* spp.	Phenotypic, PCR ***	*bla_TEM-1_*	[68]
	Farm	Chicken	Meat products	96	Healthy	*Klebsiella* spp.	Phenotypic, WGS *	*bla_CTX-M-15_*,* bla_DHA-1_*	[69]
	Slaughterhouses	Chicken	Caecal	809	Diseased	*Escherichia coli*	Phenotypic, PCR ***, WGS *	*bla_CTX-M-1_*	[70]
	Farms	Chicken	Caecal	855	Healthy	*Escherichia coli*	Phenotypic, PCR ***	*bla_CTX-M-15_*, *bla_TEM_*, *bla_SHV_*	[71]
Southern Italy	Market	Chicken, turkey	Meat	38	Healthy	*Escherichia coli*	Phenotypic	ND	[72]
	Farms	chicken, layer, turkey	Carcass, faecal	17	Healthy	*Salmonella* spp.	Phenotypic, PCR ***, WGS *	*bla_SHV-12_*	[73]
	Farms	Chicken	Meat	237	Diseased	*Escherichia coli*	PCR ***	*bla_CTX-M-1_*, *bla_CTX-M-15_*	[74]
	ND	Chicken	Meat	163	Diseased	*Escherichia coli*	Phenotypic, PCR ***	*bla_TEM-1_*, *bla_CTX-M-1_*	[75]
	ND	Chicken	Meat	145	Diseased	*Salmonella* spp.	Phenotypic	ND	[76]
	Farms	Chicken, turkey	Meat	103	Diseased	*Salmonella* spp.	Phenotypic, PCR ***, WGS *	*bla_CTX-M-1_*	[77]

* Whole genome sequencing; ** restriction fragment length polymorphism; *** polymerase chain reaction; Note: ND = not detected.

**Table 2:** Indicates all the details of antibiotic susceptibility testing, phenotypic identification, genotypic identification tests, bacterial species identified and accrediting bodies for phenotype values used in all the studies selected for this study.

Location	Sampling Date	Antibiotic Susceptibility Test	Phenotypic Confirmatory Test	Genotypic Detection Test	Accreditation Body	Bacterial Species	Reference
Whole Country	2016–2019	Disk diffusion method	Broth microdilution method	PCR	EUCAST, CLSI	*Salmonella*	[49]
	2016–2017	Broth microdilution method	Broth microdilution method	PCR	EUCAST	*Salmonella*	[50]
	2016–2017	Broth microdilution method	ND	PCR	EUCAST	*E. coli*	[51]
Northern Italy	2008–2012	Disk diffusion method	ND	PCR	CLSI	*E. coli*	[52]
	2009	Broth microdilution method	ND	PCR	EUCAST	*E. coli*	[53]
	2017–2018	ND	Double disk synergy method	PCR	CLSI	*Salmonella*	[54]
	2009	Disk diffusion method	Double disk synergy method	PCR	CLSI	*Salmonella*	[55]
	2006–2007	Disk diffusion method	Broth microdilution method	PCR	CLSI	*Salmonella*	[56]
	2010–2018	ND	Broth microdilution method	PCR	CLSI, EUCAST	*E. coli*	[57]
	2019–2021	ND	Double disk synergy method	PCR	CLSI	*E. coli*	[58]
	2017–2018	Broth microdilution method	ND	WGS	EUCAST	*Klebsiella*	[59]
	2020	ND	Double disk synergy method	PCR	CLSI	*E. coli*	[60]
Central Italy	2011–2014	Disk diffusion method	Broth microdilution method	PCR	CLSI	*Salmonella*	[61]
	2020	Disk diffusion method, broth microdilution	ND	ND	CLSI	*E. coli*	[62]
	2018	Broth microdilution method	Broth microdilution method	PCR	EUCAST	*Salmonella*	[63]
	2016–2017	Broth microdilution method	Broth microdilution method	PCR	EUCAST	*Salmonella*	[64]
	2020	Disk diffusion method	ND	WGS	EUCAST	*E. coli*	[65]
	2019	Disk diffusion method	ND	PCR	CLSI	*Salmonella*	[66]
	2016–2017	ND	ND	WGS	ND	*Salmonella*	[67]
	2023	VITEK^®^ 2 system	VITEK^®^ 2 system	PCR	CLSI	*E. coli* and *Salmonella*	[68]
	2018–2022	Kirby–Bauer method, disk diffusion method	Double disk synergy method	WGS	EUCAST	*Klebsiella*	[69]
	2017–2018	Disk diffusion method	Double disk synergy method	PCR	ND	*E. coli*	[70]
	2017–2018	Disk diffusion method	ND	PCR	EUCAST	*E. coli*	[71]
Southern Italy	2015	Disk diffusion method	ND	ND	CLSI	*E. coli*	72]
	2014–2019	Broth microdilution method	ND	WGS	EUCAST	*Salmonella*	[73]
	2013–2015	ND	Double Disk Synergy Method	PCR	EUCAST	*E. coli*	[74]
	2014–2015	Disk diffusion method	Double Disk Synergy Method	PCR	ND	*E. coli*	[75]
	2019–2021	Kirby–Bauer method	ND	ND	CLSI	*Salmonella*	[76]
	2017–2020	Broth microdilution method	ND	WGS	EUCAST	*Salmonella*	[77]

Note: WGS = whole genome sequencing, RFLP = restriction fragment length polymorphism, PCR = polymerase chain reactions, CLSI = Clinical and Laboratory standards Institute, EUCAST= European Committee on Antimicrobial Susceptibility Testing, ND = not detected.

## Data Availability

Special thanks to the whole staff of the Sede Territoriale di Brescia of IZSLER for their support.

## Supplementary Materials

The following supporting information can be downloaded at https://www.mdpi.com/article/10.3390/ani15111598/s1. [Tables S1–S3], Table S1 presents the various keywords and key platforms used to search the literature as well as the vigilance and AMR and monitoring in Italy, Table S2 represents the ESBL proportion, multidrug Resistance and bacterial species identification methods in Italian studies. Table S3 reports the excluded studies from the manuscript with the references. [112,113,114,115,116,117,118,119,120,121,122,123,124,125,126,127,128,129,130,131,132,133,134,135,136,137,138,139,140,141,142,143,144,145,146,147,148,149,150,151,152,153,154,155,156,157,158,159,160,161,162,163,164,165,166,167,168,169,170,171,172,173,174,175,176,177,178,179,180,181,182,183,184,185,186,187,188,189,190,191,192,193,194,195,196,197,198,199,200,201,202,203,204,205,206,207,208,209,210,211,212,213,214,215,216,217].

## Abbreviations

The following abbreviations are used in this manuscript:

ESBL	Extended-Spectrum Beta Lactamases
*E. coli*	*Escherichia coli*
*bla_CTX-M_*	Cefotaxime-Mediated Beta-Lactamase gene
RFLP	Restriction fragment length polymerase
PCR	Polymerase chain reaction
WGS	Whole genome sequence
CLSI	Clinical and Laboratory Standard Institute
EUCAST	European Committee on Antimicrobial Susceptibility Testing
ND	Not detected
